# Television viewing, food preferences, and food habits among children: A prospective epidemiological study

**DOI:** 10.1186/1471-2458-11-311

**Published:** 2011-05-13

**Authors:** Helle Hare-Bruun, Birgit M Nielsen, Peter L Kristensen, Niels C Møller, Per Togo, Berit L Heitmann

**Affiliations:** 1 Research Unit for Dietary Studies, Institute of Preventive Medicine, Copenhagen University Hospital, Centre for Health and Society, DK-1357 Copenhagen K, Denmark; 2 Institute of Sports Science and Clinical Biomechanics, University of Southern Denmark, DK-5230 Odense M, Denmark

## Abstract

**Background:**

Obesity has increased since the early 1980s, and despite numerous attempts, effective strategies to counter this worldwide epidemic are lacking. Food preferences are established early in life and are difficult to change later. There is therefore a need to identify factors that influence the development of food preferences. Our aim was therefore, to investigate cross-sectional and prospective associations between TV viewing habits and food preferences and habits, respectively. We hypothesized that more TV viewing was associated with less healthy concomitant and future food preferences and food habits.

**Methods:**

Data are from the Danish part of European Youth Heart Study (EYHS) I and II, a prospective cohort study conducted among 8-10-year-old and 14-16-year-old Danes in 1997-98. Six years later 2003-04 the 8-10-year-olds were followed up at age 14-16 years, and a new group of 8-10-year olds were included. Data were analysed using mixed linear regression analysis. Cross-sectional analyses included 697 8-10-year-olds and 495 14-16-year-olds. Prospective analyses included 232 pupils with complete data at baseline and follow-up. Associations between TV viewing habits and the sum of healthy food preferences (ΣHFP), and the sum of healthy food habits (ΣHFH), respectively, were examined.

**Results:**

Inverse cross-sectional associations between TV viewing (h/day) and both ΣHFP and ΣHFH were present for both the 8-10-year-old and the 14-16-year-old boys and girls. The frequency of meals in front of the TV (times/week) was also inversely associated with ΣHFP among 8-10-year-old boys, and with ΣHFH in all sex- and age groups. Among girls, baseline TV viewing (h/day) was directly associated with adverse development in the ΣHFP during follow-up. The concomitant 6-year changes in ΣHFH and TV viewing (h/day) were inversely associated in boys.

**Conclusions:**

Long time spent on TV viewing, and possibly to a lesser degree, frequent consumption of meals during TV viewing, seem to be associated with generally having unhealthy food preferences and food habits among school-aged children. These associations, however, were not generally persistent after 6 years of follow-up.

## Background

The prevalence of obesity has increased dramatically since the early 1980s [1-3], and despite numerous attempts, effective strategies to counter this worldwide epidemic are still lacking.

From birth we are genetically predisposed to a preference for the sweet and salty tastes, and an aversion against sour and bitter tastes. Food neophobia is also a genetic predisposition, but preference for novel foods can be learned and modified by repeated experience [4]. Food preferences and habits are established through experience and learning in the first years of life, which shape the later preferences and habits related to food intake [5]. Food habits of children from 3-4 years of age have been found to influence food habits through childhood and into adulthood [6,7]. Furthermore, food preferences are established early in life and are difficult to change later [4]; additionally, food habits have been found to depend largely on food preferences [8,9]. Therefore, it is essential that healthy food preferences are encouraged and promoted from early childhood in order to establish healthy food habits later in life. However, there is a need to identify factors that influence the development of food preferences to be able to apply targeted initiatives for development of healthy food preferences. Different aspects of TV viewing have been related to food habits and food preferences in children [10-16]. A number of studies have focused on the effect of advertisements on different aspects of food preferences [12,17,18]. However, the mere time spent on TV viewing, or meals eaten during TV viewing may be equally important. TV viewing offers a surplus of time for food consumption, and a few studies have found that high levels of TV viewing were associated with reduced nutritional quality in the diet of school-aged children [13-15]. Coon et al. [16] found that a large proportion of the foods eaten during TV viewing were of low nutritional value, and Blass et al. [19] that TV viewing during meals increased energy intake. Finally, Husby et al. [20] observed, in a qualitative study, that children with less healthy eating habits ate meals and snacks alone, often in front of the TV, more often than children with healthier eating habits.

Our aim was to investigate the cross-sectional associations between TV viewing habits and food preferences, and food habits, respectively; and whether TV viewing habits, and changes in TV viewing habits over the following 6 years were associated with 6-year changes in food preferences, and food habits, respectively. We hypothesized that more hours of TV viewing per day, and a higher number of meals eaten during TV viewing were associated with less healthy concomitant and future food preferences and food habits.

## Methods

### Subjects

The analyses were based on data from the Danish part of the European Youth Heart Study (EYHS) I and II, which was designed to study cardiovascular disease risk factors in children and adolescents [21]. The data were collected in 1997-98 (EYHS I) and 2003-04 (EYHS II) and both studies included 8-10-year-olds (3^rd ^grade) and 14-16-year-olds (9^th ^grade) from randomly sampled schools in the municipality of Odense, the third largest city in Denmark. The schools were stratified according to size, socioeconomic character of their uptake area, and geographical location (urban/rural). A sample of 1429 pupils was drawn from 25 schools in 1997-98 and 1019 agreed to participate. From this population 505 participants had complete data for the present study. In 2003 a new cohort of 8-10-year-olds was sampled, and the children aged 8-10 years in 1997-98 were followed up (now 14-16 years old). Of the 1480 invited in 2003-04 902 agreed to participate in EYHS II [22]. From this population 687 participants had complete data for the present study. The sampled cohorts are considered to be representative for the two age groups in Odense, but not necessarily all over Denmark.

The study was approved by the local ethics committee for Vejle and Funen (J.nr. VF 20030067) and is in accordance with the Helsinki Declaration.

### Dropout

The dropout rate from EYHS I to EYHS II was 34.9%. There was no significant difference in dropout rate between boys and girls (*P *= 0.10). However, dropout analyses were made separately for boys and girls, since most of the statistical analyses were stratified by sex. No significant differences in baseline characteristics were observed between participants and non-participants regarding ΣHFP (girls, *P *= 0.97; boys, *P *= 0.97), ΣHFH (girls, *P *= 0.70; boys, *P *= 0.76), BMI z-score (girls, *P *= 0.88; boys, *P *= 0.09), or SES (girls, *P *= 0.19; boys, *P *= 0.41) among either girls or boys. Boys who participated in EYHS I but not in EYHS II watched more hours of TV per day (*P *= 0.05), and they more frequently watched TV during meals (*P *= 0.003).

### Measurements

#### Food preferences and food habits

The outcome variable "sum of healthy food preferences (ΣHFP)" was based on nine questions from a validated [23] computer-based questionnaire. The questionnaire was developed specifically for EYHS and has been shown to be valid and reliable for both 8-10-year-olds and 14-16-year-olds [21]. The children answered the questionnaire individually in a quiet room without interaction with other children. Both age groups answered the same questionnaire. A researcher, who was instructed not to interfere with the children, was present in case of questions or uncertainties during completion of the questionnaires. The children had to answer each question before the next question appeared on the screen; thereby the amount of missing data was reduced [24]. The children were asked about their preference for French fries, carbonated drinks, fruit, vegetables, pizza, salad, crisps, burgers, and sweets/chocolate, with answers given on a 3-point scale ('I like it', 'it's OK', 'I don't like it'). The healthy items (fruit, vegetables and salad) were given 2, 1, and 0 points for the answers 'I like it', 'it's OK', and 'I don't like it', respectively. Conversely, the unhealthy items (French fries, carbonated drinks, pizza, crisps, burgers, and sweets/chocolate) were given 0, 1, and 2 points for the answers 'I like it', 'it's OK', and 'I don't like it', respectively. The 9 scores were then summed to create the variable "sum of healthy food preferences" resulting in a variable with a theoretical range of 0-18, where a low score indicates unhealthy food preferences and a high score indicates healthy food preferences.

Likewise, the outcome variable "sum of healthy food habits (ΣHFH)" was based on 9 questions regarding the children's' intake of French fries, carbonated drinks, fruit, vegetables, pizza, salad, crisps, burgers, and sweets/chocolate (the same foods as the ΣHFP variable was based on). Answers on intake habits were given on a 4-point scale ('every day', 'almost every day', '1-2 times a week', 'almost never or never'). The healthy food items (the same as for ΣHFP) were given 3, 2, 1, and 0 points for the answers 'every day', 'almost every day', '1-2 times a week', and 'almost never or never', respectively, and the unhealthy food items were given 0, 1, 2, and 3 point for the answers 'every day', 'almost every day', '1-2 times a week', and 'almost never or never', respectively. The 9 scores were summed to create the variable "sum of healthy food habits" with a theoretical range of 0-27. Again, low scores indicated less healthy food habits than high scores.

#### TV viewing

The exposure variables were 'TV viewing during meals (times/week)' and 'TV viewing (hours/day)'. These variables were likewise self-reported from the previously described computer-based questionnaire. The variable 'TV viewing during meals' was based on the question "How often do you watch TV while you eat?" It was measured in 5 categories: 'every day', 'most days', 'once or twice a week', 'only on weekends', and 'almost never or never'. Due to the limited number of participants, and a slightly skewed distribution of the variable, the 5 categories were collapsed to 3 categories: 'every day or most days', 'once or twice a week or only on weekends', and 'almost never or never'. Two questions on hours of TV viewing, before (0, <1-2, and >2 hours) and after school (0, <1, 1-2, 2-3, and >3 hours), respectively, were recoded so that for TV viewing before school 0 h = 0 h, <1 h = 0.5 h, 1-2 h = 1.5 h, and >2 h = 2.5 h and for TV viewing after school 0 h = 0 h, <1 h = 0.5 h, 1-2 h = 1.5 h, 2-3 h = 2.5 h, and >3 h = 3.5 h. The two recoded variables were then added to form the variable 'TV viewing (h/day)'. TV viewing (h/day) was split into 3 categories (<1, 1-2, and >2 h/day) based on the distribution of the variable in the study population in order to obtain three groups with approximately equal n.

#### Covariates

Body weight was measured to the nearest 100 g with a beam-scale weight with the subjects wearing light clothing only, according to a standardized protocol [21,25]. Height was measured to the nearest half centimetre using a stadiometer. BMI was calculated as body weight (kg) divided by height (m) squared, and converted to z-scores using Danish age- and sex specific reference values [26]. We chose to use the BMI z-score, as this variable is independent of sex and age [27], and comparison between sex and age groups is therefore much more straight forward than when using the original BMI variable. Maternal and paternal BMI were calculated from self reported height and weight.

Physical activity was measured with MTI Actigraph accelerometer (Manufacturing Technology, Fort Walton Beach, Florida, USA). The procedure has been described in detail elsewhere [21,28]. In brief, the accelerometers were designed to measure and record vertical body accelerations. They were worn on the waist. The participants were instructed to wear the accelerometers for at least 5 consecutive days including weekend days, except during water-based activities and while they were sleeping. The activity counts were averaged and stored every 60 seconds. If the record showed zero activity for a period of 10 minutes or more it was assumed that the accelerometer had been taken off, and the data for the period were discarded. A recording was considered successful if the accelerometer had been worn at least 10 hours per day for 3 days [28].

Socioeconomic status (SES) was based on questionnaire information about maternal education, which has been shown to be the best indicator of SES in children [29]. It was categorized in 6 groups; elementary school (8^th^-10^th ^grade), high school education, vocational education, short further education, medium further education, and long further education. The 6 categories were reduced to two categories by collapsing the first three into the category 'no further education' and the last three into the category 'some further education'.

Information on diet was collected using a single 24 h recall supplemented with a qualitative food record for each child. The qualitative food records were completed at home the day before a face-to-face interview. All interviews were carried out on school days, and the same person interviewed all children in 1997-98. In 2003-04 all 8-10-year-olds were interviewed by one interviewer, and all 14-16-year-olds were interviewed by another interviewer. Quantities were estimated using common household measures and food pictures. Dietary intakes were entered into a database for calculation of nutrient intakes according to the Danish food composition tables [30].

### Statistical analyses

We collapsed data on from EYHS I and EYHS II in the cross-sectional analyses in order to increase power. The cross-sectional analyses were performed separately for 8-10-year-olds and 14-16-year-olds, respectively.

Differences in descriptive characteristics between boys and girls were tested in multiple regression analysis (proc glm in SAS, version 9.1, SAS Institute Inc., Cary, NC, USA) with adjustment for survey year (continuous variables) or with Chi-square test (categorical variables).

We used multiple regression analysis for the main analyses (proc mixed in SAS). School was included in the models as a random effect to counterbalance the effect of clustering within schools caused by the cluster-sampling design. The regression analyses were done both with and without adjustment for potential confounders. Confounders were chosen *a priori *and were kept in the analyses even if they did not change the estimates. We stratified all analyses by sex due to interactions between sex and TV viewing (h/day), and sex and TV viewing during meals (times/week) both in analyses of food preferences and in analyses of food habits (All *P *< 0.0001).

All cross-sectional analyses were adjusted for survey year and the adjusted analyses were further adjusted for BMI z-score, physical activity (mean counts per minute), maternal BMI, paternal BMI, and SES. Participants who had missing data on one or more of the variables used for the main analyses were excluded.

Associations between 6-year changes in ΣHFP, and ΣHFH and baseline TV viewing (h/day), and TV viewing during meals (times/week) were examined in the prospective analyses. These analyses were adjusted for baseline values of BMI z-score, physical activity (mean counts per minute), maternal BMI, paternal BMI, SES, and the baseline value of the outcome variable (ΣHFP or ΣHFH). Associations between 6-year changes in ΣHFP, and ΣHFH and 6-year changes in TV viewing habits (TV viewing (h/day), and TV viewing during meals (times/week)) were examined by including both baseline and follow-up values of TV viewing habits in the models. When adjusted for baseline, the estimate for the follow-up variable is a measure of the change from baseline to follow-up. These analyses were, likewise, further adjusted for baseline values of BMI z-score, physical activity (mean counts per minute), maternal BMI, paternal BMI, SES, and the baseline value of the outcome variable (ΣHFP or ΣHFH).

As mentioned, the dietary assessment method used in this study was a single 24 h recall. This method provides valid data on habitual dietary intake on the group level [31], but not on the individual level. In order to assess the agreement between the ΣHFH variable and more traditional dietary variables and check the internal validity of the ΣHFH variable we therefore calculated mean values of fat energy percentage (E%), added sugar E%, liquid sugar (sugar from carbonated drinks and lemonade) E%, and liquid sugar (g) by tertiles of ΣHFH based on dietary data from the 24 h recalls. Subsequently, linear trend analyses, between tertiles of ΣHFH and the dietary variables were carried out.

## Results

The cross-sectional analyses included 382 8-10-year-old girls (176 from the 1997-98 survey and 206 from the 2003-04 survey), 315 8-10-year-old boys (150 from the 1997-98 survey and 165 from the 2003-04 survey), 275 14-16-year-old girls (97 from the 1997-98 survey and 178 from the 2003-04 survey), and 220 14-16-year-old boys (82 from the 1997-98 survey and 138 from the 2003-04 survey) with complete data. The prospective analyses included 131 girls and 101 boys with complete data at both baseline and follow-up.

Table 1 shows descriptive baseline characteristics of the participants. Mean TV viewing time increased in the 8-10-year-olds from 1.23 h/day (1 h, 14 min) in 1997/98 to 1.36 h/day (1 h, 22 min) in 2003/04, and in the 14-16-year-olds from 1.61 h/day (1 h, 37 min) in 1997/98 to 1.70 h/day (1 h, 42 min) in 2003/04. The correlation between ΣHFP and ΣHFH (Pearson's *r*) was 0.36 (*P *< 0.0001) in the 8-10-year-olds, and 0.46 (*P *< 0.0001) in the 14-16-year-olds. Boys generally had poorer food habits and preferences than girls and they also watched more TV.

**Table 1:** Selected characteristics of participants

	*8-10-year-olds*					*14-16-year-olds*				
	Girls (n = 382)		Boys (n = 315)		*P*	Girls (n = 275)		Boys (n = 220)		*P*
*Continuous variables*^1^										
Age (years)	9.7	(0.4)	9.8	(0.4)	**<0.0001**	15.6	(0.4)	15.7	(0.4)	0.07
Height (cm)	139.2	(6.4)	140.3	(6.5)	**0.03**	165.5	(6.6)	176.4	(7.3)	**<0.0001**
Bodyweight (kg)	33.4	(6.2)	34.1	(6.3)	0.19	57.9	(9.6)	65.5	(10.5)	**<0.0001**
BMI z-score^2^	0.3	(1.1)	0.3	(1.2)	0.83	0.4	(1.0)	0.5	(1.0)	**0.04**
Maternal BMI (kg/m^2^)	23.5	(3.9)	24	(4.2)	0.09	23.8	(4.5)	24.6	(5.4)	**0.05**
Paternal BMI (kg/m^2^)	25.4	(3.3)	25.4	(2.9)	0.83	25.7	(3.5)	25.6	(3.3)	0.77
Energy intake (MJ)^3^	8.4	(2.2)	9.4	(2.7)	**<0.0001**	8.5	(2.9)	12.0	(4.3)	**<0.0001**
Fat E%^3^	31.2	(7.2)	31.5	(6.9)	0.73	28.4	(8.1)	30.5	(7.6)	**0.006**
Sugar E%^3^	9.5	(6.6)	9.7	(6.9)	0.65	9.5	(8.0)	10.7	(7.1)	0.11
Liquid sugar (E%)^3^	3.5	(4.8)	3.7	(4.7)	0.53	3.9	(5.9)	5.6	(6.1)	**0.002**
Liquid sugar (g/day)^3^	16.5	(21.5)	20.8	(26.3)	**<0.0001**	20.3	(35.9)	40.4	(46.3)	**<0.0001**
Σ healthy food preferences	7.2	(2.5)	6.4	(2.4)	**<0.0001**	7.6	(2.4)	6.1	(2.3)	**<0.0001**
Σ healthy food habits	19.9	(3.1)	19.1	(3.3)	**0.003**	20.6	(2.9)	18.3	(3.3)	**<0.0001**
TV viewing (h/day)	1.2	(0.9)	1.4	(1.1)	**0.0005**	1.5	(1.1)	1.8	(1.2)	**0.01**
Physical activity (mean counts/min)	592.1	(189.3)	710.3	(217.8)	**<0.0001**	410.6	(143.9)	477.4	(171.3)	**<0.0001**
										
*Categorical variables*^4^										
SES										
Low	186	(48.7)	153	(48.6)		131	(47.6)	115	(52.3)	
High	196	(51.3)	162	(51.4)	0.97	144	(52.4)	105	(47.7)	0.31
TV viewing										
<1 h/day	162	(42.4)	115	(36.5)		85	(30.9)	58	(26.4)	
1-2 h/day	180	(47.1)	145	(46.0)		123	(44.7)	93	(42.3)	
>2 h/day	40	(10.5)	55	(17.5)	**0.02**	67	(24.4)	69	(31.4)	0.20
TV meals										
Almost never or never	92	(24.1)	81	(25.7)		54	(19.6)	50	(22.7)	
1-2 times a week	171	(44.8)	98	(31.1)		111	(40.4)	64	(29.1)	
Every day or most days	119	(31.2)	136	(43.2)	**0.0005**	110	(40.0)	106	(48.2)	**0.03**

^1^*P *for difference between boys and girls of same age, tests for difference in multiple regression analysis. Analyses were adjusted for survey year. Values are mean (SD).

^2^Based on a Danish reference population [26].

^3^Due to some missing values on the dietary variables these values are based on 356 8-10-year-old girls, 278 8-10-year-old boys, 265 14-16-year-old girls, and 208 14-16-year-old boys.

^4^*P *for difference between boys and girls of same age, Chi-square test for difference between groups. Values are n (%).

SD, standard deviation; BMI, body mass index; MJ, Mega Joule; E%, percentage of energy; SES, socioeconomic status.

Dietary fat E%, total added sugar E%, liquid sugar E%, and liquid sugar intake (g) all decreased across increasing tertiles of ΣHFH in 8-10-year-old girls and boys. In 14-16-year-old girls dietary fat E%, and total added sugar E% likewise decreased across tertiles of ΣHFH, and in 14-16-year-old boys total added sugar E%, liquid sugar E% and liquid sugar intake (g) decreased across tertiles of ΣHFH, table 2.

**Table 2:** Mean values of selected dietary variables according to tertiles of Σ healthy food habits^1^

	*Tertiles of Σ Healthy food habits*			
	1	2	3	*P*_trend_^3^
*Girls, 8-10 years*				
n	108	135	113	
Fat E%	32.2 (6.9)^2^	31.3 (7.3)	30.3 (7.2)	**0.05**
Added sugar E%	10.8 (7.4)	9.5 (6.3)	8.2 (6.0)	**0.003**
Liquid sugar E%	4.6 (5.4)	3.4 (4.6)	2.4 (4.0)	**0.0005**
Liquid sugar (g)	20.9 (22.1)	17.0 (22.1)	11.6 (19.3)	**0.001**
				
*Boys, 8-10 years*				
n	108	82	88	
Fat E%	32.2 (6.8)	32.5 (6.8)	29.7 (6.9)	**0.02**
Added sugar E%	10.7 (7.7)	9.4 (5.8)	8.8 (6.5)	**0.04**
Liquid sugar E%	4.5 (5.4)	3.2 (3.9)	3.2 (4.3)	**0.05**
Liquid sugar (g)	25.4 (31.4)	18.9 (22.6)	17.1 (21.5)	**0.02**
				
*Girls, 14-16 years*				
n	87	99	79	
Fat E%	30.2 (8.9)	28.8 (7.1)	26.0 (7.7)	**0.001**
Added sugar E%	11.5 (8.5)	8.6 (6.8)	8.4 (8.4)	**0.008**
Liquid sugar E%	4.8 (5.7)	3.4 (5.4)	3.6 (6.6)	0.16
Liquid sugar (g)	24.2 (28.3)	17.4 (28.4)	19.7 (49.3)	0.37
				
*Boys, 14-16 years*				
n	76	72	60	
Fat E%	31.1 (6.8)	31.4 (8.9)	28.7 (6.7)	0.11
Added sugar E%	12.9 (7.6)	9.8 (7.3)	8.9 (5.6)	**0.0006**
Liquid sugar E%	7.5 (7.0)	4.8 (5.6)	4.2 (4.8)	**0.0007**
Liquid sugar (g)	58.0 (55.3)	31.8 (38.9)	28.3 (34.2)	**<0.0001**

^1^Due to some missing values on the dietary variables these analyses include 356 8-10-year-old girls, 278 8-10-year-old boys, 265 14-16-year-old girls, and 208 14-16-year-old boys. Uneven numbers in the 3 tertiles are due to the properties of the ΣHFH variable, which includes whole numbers only. Higher values of ΣHFH indicate healthier food habits.

^2^Mean (SD), all such values.

^3^*P*-value for linear trend across tertiles of ΣHFH

E%, percentage of energy

### Food preferences

More TV viewing (hours/day) was associated with lower ΣHFP in all 8-10-year-olds and in 14-16-year-old girls, table 3. Among girls, more hours of TV viewing per day at age 8-10 years was inversely associated with the subsequent 6-year change in ΣHFP. The 6-year change in TV viewing (h/day) was not associated with the 6-year change in ΣHFP among girls. No associations between either baseline TV viewing (h/day) and the 6-year change in ΣHFP, or the 6-year change in TV viewing (h/day) and the 6-year change in ΣHFP was observed among boys, table 4.

**Table 3:** Cross-sectional associations between ΣHFP, and ΣHFH and TV viewing habits in 8-10-year-olds and 14-16-year-olds

	*8-10 years*				*14-16 years*			
	
	Girls (n = 382)		Boys (n = 315)		Girls (n = 275)		Boys (n = 220)	
Σ Healthy Food Preferences								
**TV viewing (h/day)**								
*Crude model*^2^								
>2 h/day	-0.94*	(-1.79; -0.08)^1^	-1.31***	(-2.07; -0.55)	-0.96*	(-1.71; -0.21)	-0.75	(-1.54; 0.04)
1-2 h/day	-0.54*	(-1.06; -0.01)	-1.11***	(-1.70; -0.53)	-0.50	(-1.15; 0.15)	-0.24	(-0.99; 0.50)
<1 h/day	0	ref	0	ref	0	ref	0	ref
*Adjusted model*^3^								
>2 h/day	-1.06*	(-1.91; -0.21)	-1.36***	(-2.13; -0.59)	-0.94*	(-1.69; -0.18)	-0.78	(-1.57; 0.02)
1-2 h/day	-0.61*	(-1.13; -0.09)	-1.12***	(-1.70; -0.54)	-0.44	(-1.08; 0.21)	-0.38	(-1.13; 0.36)
<1 h/day	0	ref	0	ref	0	ref	0	ref
								
**TV meals/week**								
*Crude model*^2^								
most days/every day	-0.04	(-0.71; 0.63)	-0.83*	(-1.50; -0.16)	-0.60	(-1.37; 0.17)	-0.67	(-1.43; 0.10)
1-2 times/week	0.68*	(0.06; 1.31)	-0.29	(-1.01; 0.42)	-0.26	(-1.02; 0.50)	-0.68	(-1.52; 0.15)
almost never/never	0	ref	0	ref	0	ref	0	ref
*Adjusted model*^3^								
most days/every day	-0.04	(-0.71; 0.63)	-0.84*	(-1.52; -0.16)	-0.66	(-1.44; 0.12)	-0.72	(-1.48; 0.05)
1-2 times/week	0.60	(-0.02; 1.22)	-0.28	(-1.00; 0.44)	-0.33	(-1.10; 0.44)	-0.72	(-1.56; 0.12)
almost never/never	0	ref	0	ref	0	ref	0	ref
Σ Healthy Food Habits								
								
**TV viewing (h/day)**								
*Crude model*^2^								
>2 h/day	-1.45**	(-2.50; -0.40)	-1.67**	(-2.71; -0.64)	-1.47**	(-2.37; -0.56)	-2.16***	(-3.27; -1.04)
1-2 h/day	-0.72*	(-1.37; -0.08)	-0.73	(-1.51; 0.06)	-0.20	(-0.98; 0.59)	-0.88	(-1.93; 0.16)
<1 h/day	0	ref	0	ref	0	ref	0	ref
*Adjusted model*^3^								
>2 h/day	-1.58**	(-2.60; -0.55)	-1.48**	(-2.51; -0.45)	-1.39**	(-2.30; -0.49)	-2.02***	(-3.15; -0.89)
1-2 h/day	-0.94**	(-1.57; -0.31)	-0.67	(-1.44; 0.10)	-0.16	(-0.93; 0.62)	-0.80	(-1.87; 0.26)
<1 h/day	0	ref	0	ref	0	ref	0	ref
								
**TV meals/week**								
*Crude model*^2^								
most days/every day	-1.61***	(-2.43; -0.79)	-2.50***	(-3.36; -1.65)	-1.28**	(-2.20; -0.37)	-2.14***	(-3.20; -1.08)
1-2 times/week	-0.60	(-1.36; 0.16)	-1.00*	(-1.92; -0.07)	0.16	(-0.75; 1.07)	-0.60	(-1.77; 0.56)
almost never/never	0	ref	0	ref	0	ref	0	ref
*Adjusted model*^3^								
most days/every day	-1.56***	(-2.36; -0.76)	-2.25***	(-3.11; -1.40)	-1.24**	(-2.16; -0.32)	-2.04***	(-3.12; -0.96)
1-2 times/week	-0.71	(-1.45; 0.03)	-1.02*	(-1.93; -0.11)	0.07	(-0.85; 0.98)	-0.51	(-1.69; 0.67)
almost never/never	0	ref	0	ref	0	ref	0	ref

ΣHFP, Sum of healthy food preferences; ΣHFH, sum of healthy food habits; ref, reference category.

^1^Estimate (95% confidence interval), all such values.

^2^Adjusted for survey year. School included in model as random effect.

^3^Adjusted for survey year, BMI z-score, maternal BMI, paternal BMI, physical activity, SES (maternal education). School included in model as random effect.

*P ≤ 0.05, **P ≤ 0.01, ***P ≤ 0.001.

**Table 4:** Six-year changes in ΣHFP and ΣHFH in relation to baseline and 6-year changes in TV viewing habits

	*Δ (ΣHFP)*				*Δ (ΣHFH)*			
	
	Girls (n = 131)		Boys (n = 101)		Girls (n = 131)		Boys (n = 101)	
*TV (h/day) at baseline*^2^								
>2 h/day	-1.58*	(-3.10; -0.06)^1^	0.18	(-1.04; 1.39)	-1.01	(-2.80; 0.79)	-1.51	(-3.54; 0.52)
1-2 h/day	-0.57	(-1.47; 0.32)	-0.44	(-1.25; 0.38)	0.02	(-1.04; 1.08)	-0.53	(-1.90; 0.85)
<1 h/day	0	ref	0	ref	0	ref	0	ref
								
*TV (h/day) at follow-up adjusted for TV (h/day) at baseline*^3^								
>2 h/day	0.10	(-1.07; 1.26)	0.07	(-0.99; 1.14)	-0.85	(-2.20; 0.51)	-2.62*	(-4.30; -0.94)
1-2 h/day	0.26	(-0.75; 1.27)	0.07	(-0.96; 1.11)	-0.38	(-1.56; 0.80)	-1.28	(-2.88; 0.33)
<1 h/day	0	ref	0	ref	0	ref	0	ref
								
*TV during meals (times/week) at baseline*^2^								
most days/every day	-0.55	(-1.68; 0.58)	0.31	(-0.75; 1.36)	0.60	(-0.73; 1.93)	-1.07	(-2.83; 0.69)
1-2 times/week	-0.28	(-1.34; 0.77)	0.55	(-0.47; 1.58)	0.64	(-0.58; 1.87)	-0.98	(-2.71; 0.75)
almost never/never	0	ref	0	ref	0	ref	0	ref
								
*TV during meals (times/week) at follow-up adjusted for TV during meals (times/week) at baseline*^3^								
most days/every day	-0.10	(-1.29; 1.10)	0.32	(-0.86; 1.50)	-0.61	(-1.98; 0.76)	-0.75	(-2.73; 1.23)
1-2 times/week	0.17	(-1.04; 1.38)	0.20	(-0.97; 1.37)	0.65	(-0.73; 2.03)	0.06	(-1.89; 2.02)
almost never/never	0	ref	0	ref	0	ref	0	ref

ΣHFP, Sum of healthy food preferences; ΣHFH, sum of healthy food habits; Δ, change; ref, reference category.

^1^Estimate (95% confidence interval), all such values.

^2^Adjusted for baseline values of ΣHFP/ΣHFH, BMI z-score, maternal BMI, paternal BMI, physical activity, SES (maternal education). School included in model as random effect.

^3^Adjusted for baseline values of ΣHFP/ΣHFH, TV viewing (h/day or meals/week), BMI z-score, maternal BMI, paternal BMI, physical activity, SES (maternal education). School included in model as random effect.

*P ≤ 0.05.

Boys aged 8-10 years, who watched TV during meals every day or most days had less healthy food preferences than those who rarely watched TV during meals, whereas 8-10-year-old girls who watched TV during meals 1-2 times per week had higher ΣHFP than those who rarely watched TV during meals. In 14-16-year-olds no association between ΣHFP and TV viewing during meals was present, table 3. The 6-year change in the ΣHFP was not related to either baseline or 6-year change in TV viewing during meals (times/week) in boys or girls, table 4.

### Food habits

Both 8-10-year-olds and 14-16-year-olds of both sexes had less healthy food habits the more hours they spent in front of the TV per day, table 3. The 6-year change in ΣHFH was not associated with either baseline or 6-year change in TV viewing (h/day) in girls, However, the concomitant 6-year changes in ΣHFH and TV viewing (h/day) were inversely associated in boys, table 4.

Likewise, boys and girls of both age groups who watched TV during meals every day or most days had lower ΣHFH than those who rarely watched TV during meals, table 3. The 6-year change in ΣHFH was not related to baseline TV viewing during meals (times/week) or 6-year change in TV viewing during meals (times/week) in either girls or boys, table 4.

## Discussion

We examined whether high levels of TV viewing were associated with unhealthy food preferences and food habits and found for both boys and girls that the more time spent in front of the TV the poorer the food preferences and food habits, although the associations were slightly stronger in boys than girls. The finding of less healthy food preferences and food habits observed among boys in the present study, is in line with previous studies, where both preference for- and intake of fruit and vegetables have been observed to be higher among girls [32-35], whereas boys have been found to have higher preference for meat, and fatty and sugary foods [33,35]. In combination with the poorer mean food preferences and food habits among boys, the stronger associations between TV viewing habits and both food preferences, and food habits, could suggest that boys' food preferences and food habits may be more susceptible to be influenced by TV viewing than girls' food preferences and food habits.

Poorer food habits included a higher consumption of French fries, carbonated drinks, pizza, crisps, burgers, and sweets/chocolate and a lower consumption of fruit, vegetables, and salad. These results are supported by other studies, which have found high levels of TV viewing to be associated with low fruit intake, high soft drink intake and high fat intake in school-aged children [13-15]. However, previous studies did not examine the persistence over time of these relations. We examined if TV viewing habits at age 8-10 years and the 6-year changes in TV viewing habits were related to changes in food preferences and food habits during the 6 years of follow-up, but found that such associations were not consistent.

The straightforward explanation is that food preferences and food habits are established early [4] and may be very fixed even at the age of 8-10 years. The changes that might occur during the follow-up period may, for that reason, be minimal. The results of the prospective analyses therefore support the hypothesis that food preferences and food habits are established early in life, and that later changes are likely to be small. The lack of associations in the prospective analyses may, however, in part be due to the long time span between the two surveys. Trends in TV viewing habits may have changed during the follow-up period, which may have obscured potential associations. Nevertheless, we found only minimal increases in total TV viewing time (h/day) in both age groups in 2003 compared to 1997. Hence, potential confounding by differences in TV viewing would have to be in the quality of the TV programs, or in the behaviour associated with TV viewing. Unfortunately, we did not have information about these factors.

The outcome measures ΣHFP and ΣHFH were created because we wanted a measure of food preferences, and also a comparable measure of food habits. We compared ΣHFH with fat intake, and sugar intake and found good agreement between ΣHFH and the dietary variables. Even though the nutrient intake was assessed for one day only, we therefore believe that the ΣHFH is a reasonable indicator of the children's food habits. It is not possible to evaluate the ΣHFP variable in the same way since food preferences are subjective and only partly related to the food habits.

The sums of healthy food preferences and healthy food habits were, however, positively correlated. The correlations were moderate, though, indicating that factors other than preference influence food intake habits, even in childhood. One such factor could be parental control. A high level of parental control of a child's eating habits has been found to result in food preferences that were opposite to those intended by the parents. For instance, it has been observed that children who were pressured to eat specific foods to get a reward tended to develop an aversion against these foods [36,37]. In line with this, other studies have found that restricted foods often became preferred foods [38,39]. Food preferences and actual food intake is therefore not necessarily highly correlated. The correlation between food preferences and food habits was slightly stronger among the 14-16-year-olds than among the 8-10-year-olds. It could be speculated that the 14-16-year-olds experience less parental control of their eating habits than the 8-10-year-olds, which may contribute to the observed stronger correlation in this age group. It seems plausible that younger children have less influence on their intake and hence, associations between food preferences and habits in this age group would be expected to be weaker than in older age groups.

TV viewing measured as hours per day was more consistently associated with food preferences than the frequency of TV viewing during meals. This may be related to a potentially less accurate measurement of the frequency of TV viewing during meals than the hours of TV viewing per day, since the question regarding TV viewing during meals may have been less clear than the questions regarding the number of hours of TV viewing per day. The participants were asked, "How often do you watch TV while you eat?" It was not specified whether this included all foods or only regular meals. However, we reduced the original five categories to three categories before analyzing the data, and potential misclassifications are therefore likely to have been reduced. Furthermore the reliability and validity of the questionnaire has been tested and found to be good in both age groups [21].

Part of the effect of TV viewing on food preferences and food habits is likely to be mediated through TV commercials. Previous studies have found that roughly 30-50% of the TV commercials directed towards children are for foods high in sugar and/or fat [40-44], and several studies report that children prefer advertised food items over non-advertised food items [10-12]. Unfortunately, no information on TV commercials was available and thus the effect of commercials could not be taken into account in the analyses.

A few other limitations should be noted. First, only 232 participants had complete information for prospective analyses, mainly due to missing information on physical activity measured by accelerometer. Exclusion of physical activity from the analyses gave essentially similar results but increased the sample size by 50% (data not shown). Hence, type II error is unlikely to be the reason for the lack of associations in the prospective analyses.

Second, the analyses were based on questionnaire variables and a potential risk of misreporting is present. However, a random bias in the reporting of TV viewing and/or food preferences and -habits would tend to attenuate the true associations and hence, results may have been stronger than those observed.

## Conclusions

The results from this study indicate that both food preferences and food habits are associated with TV viewing habits cross-sectionally. Time spent on TV viewing, and to a lesser degree TV viewing during meals, were associated with poorer food preferences and food habits among school-aged children. Seen from a public health perspective, interventions aiming at improving food preferences and food habits in children should, therefore, consider TV viewing as a potential important influencing factor, and restrictions in especially total TV viewing time may be beneficial for the development of healthy food preferences and food habits. Compared to girls, boys seemed to prefer and eat less healthy foods in general. These associations, however, were not generally persistent after 6 years of follow-up.

## Competing interests

The authors declare that they have no competing interests.

## Authors' contributions

PLK and NCM participated in the data collection. HH-B and BLH were responsible for the conception and design of the study. HH-B analysed the data and drafted the manuscript. BMN and BLH contributed to the analysis and interpretation of data. HH-B, BMN, PLK, NCM, PT, and BLH edited and reviewed the manuscript. All authors read and approved the final manuscript.

## Pre-publication history

The pre-publication history for this paper can be accessed here:

http://www.biomedcentral.com/1471-2458/11/311/prepub

## References

[B1] Berghofer A Pischon T Reinhold T Apovian C Sharma A Willich S Obesity prevalence from a European perspective: a systematic review BMC Public Health 2008 11 200 10.1186/1471-2458-8-200 18533989 PMC2441615

[B2] Flegal KM Epidemiologic aspects of overweight and obesity in the United States Physiology & Behavior 2005 11 599 602 10.1016/j.physbeh.2005.08.050 16242735

[B3] Hedley AA Ogden CL Johnson CL Carroll MD Curtin LR Flegal KM Prevalence of Overweight and Obesity Among US Children, Adolescents, and Adults, 1999-2002 JAMA 2004 11 2847 2850 10.1001/jama.291.23.2847 15199035

[B4] Birch LL Development of food preferences Annu Rev Nutr 1999 11 41 62 10.1146/annurev.nutr.19.1.41 10448516

[B5] Birch LL Psychological influences on the childhood diet J Nutr 1998 11 407S 410S 9478037 10.1093/jn/128.2.407S

[B6] Singer MR Moore LL Garrahie EJ Ellison RC The tracking of nutrient intake in young children: the Framingham Children's Study Am J Public Health 1995 11 1673 1677 10.2105/AJPH.85.12.1673 7503343 PMC1615722

[B7] Kelder SH Perry CL Klepp KI Lytle LL Longitudinal tracking of adolescent smoking, physical activity, and food choice behaviors Am J Public Health 1994 11 1121 1126 10.2105/AJPH.84.7.1121 8017536 PMC1614729

[B8] Fisher JO Birch LL Fat preferences and fat consumption of 3- to 5-year-old children are related to parental adiposity J Am Diet Assoc 1995 11 759 764 10.1016/S0002-8223(95)00212-X 7797805

[B9] Resnicow K vis-Hearn M Smith M Baranowski T Lin LS Baranowski J Social-cognitive predictors of fruit and vegetable intake in children Health Psychol 1997 11 272 276 9152706 10.1037//0278-6133.16.3.272

[B10] Borzekowski DL Robinson TN The 30-second effect: an experiment revealing the impact of television commercials on food preferences of preschoolers J Am Diet Assoc 2001 11 42 46 10.1016/S0002-8223(01)00012-8 11209583

[B11] Halford JC Boyland EJ Cooper GD Dovey TM Smith CJ Williams N Children's food preferences: Effects of weight status, food type, branding and television food advertisements (commercials) Int J Pediatr Obes 2008 11 31 38 10.1080/17477160701645152 17963122

[B12] Robinson TN Borzekowski DL Matheson DM Kraemer HC Effects of fast food branding on young children's taste preferences Arch Pediatr Adolesc Med 2007 11 792 797 10.1001/archpedi.161.8.792 17679662

[B13] Haerens L Craeynest M Deforche B Maes L Cardon G De Bourdeaudhuij I The contribution of psychosocial and home environmental factors in explaining eating behaviours in adolescents Eur J Clin Nutr 2007 11 51 59 17299461 10.1038/sj.ejcn.1602681

[B14] Grimm GC Harnack L Story M Factors associated with soft drink consumption in school-aged children J Am Diet Assoc 2004 11 1244 1249 10.1016/j.jada.2004.05.206 15281041

[B15] Coon KA Goldberg J Rogers BL Tucker KL Relationships between use of television during meals and children's food consumption patterns Pediatrics 2001 11 E7 10.1542/peds.107.1.e7 11134471

[B16] Coon KA Tucker KL Television and children's consumption patterns. A review of the literature Minerva Pediatr 2002 11 423 436 12244280

[B17] Halford JC Boyland EJ Hughes G Oliveira LP Dovey TM Beyond-brand effect of television (TV) food advertisements/commercials on caloric intake and food choice of 5-7-year-old children Appetite 2007 11 263 267 10.1016/j.appet.2006.12.003 17258351

[B18] Halford JC Gillespie J Brown V Pontin EE Dovey TM Effect of television advertisements for foods on food consumption in children Appetite 2004 11 221 225 10.1016/j.appet.2003.11.006 15010186

[B19] Blass EM Anderson DR Kirkorian HL Pempek TA Price I Koleini MF On the road to obesity: Television viewing increases intake of high-density foods Physiol Behav 2006 11 597 604 10.1016/j.physbeh.2006.05.035 16822530

[B20] Husby I Heitmann BL O'Doherty JK Meals and snacks from the child's perspective: the contribution of qualitative methods to the development of dietary interventions Public Health Nutr 2008 1 9 10.1017/S1368980008003248 18671890

[B21] Riddoch C Edwards D Page A Froberg K Anderssen SA Wedderkopp N The European Youth Heart Study - Cardiovascular Disease Risk factors in Children: Rationale, Aims, Study Design, and Validation of Methods J Physical Activity Health 2005 11 115 129

[B22] Kristensen PL Korsholm L Møller NC Wedderkopp N Andersen LB Froberg K Sources of variation in habitual physical activity of children and adolescents: the European youth heart study Scand J Med Sci Sports 2008 11 298 308 10.1111/j.1600-0838.2007.00668.x 17555541

[B23] Ommundsen Y Page A Ku PW Cooper AR Cross-cultural, age and gender validation of a computerised questionnaire measuring personal, social and environmental associations with children's physical activity: the European Youth Heart Study Int J Behav Nutr Phys Act 2008 11 29 10.1186/1479-5868-5-29 18489736 PMC2430583

[B24] Ekelund U Brage S Froberg K Harro M Anderssen SA Sardinha LB TV viewing and physical activity are independently associated with metabolic risk in children: the European Youth Heart Study PLoS Med 2006 11 e488 10.1371/journal.pmed.0030488 17194189 PMC1705825

[B25] Council of Europe The Eurofit Test Battery 1988 Strasbourg, France, Council of Europe

[B26] Nysom K Mølgaard C Hutchings B Michaelsen KF Body mass index of 0 to 45-y-old Danes: reference values and comparison with published European reference values Int J Obes Relat Metab Disord 2001 11 177 184 10.1038/sj.ijo.0801515 11410817

[B27] Poskitt EM Defining childhood obesity: the relative body mass index (BMI). European Childhood Obesity group Acta Paediatr 1995 11 961 963 10.1111/j.1651-2227.1995.tb13806.x 7488831

[B28] Møller NC Kristensen PL Wedderkopp N Andersen LB Froberg K Objectively measured habitual physical activity in 1997/1998 vs 2003/2004 in Danish children: the European Youth Heart Study Scand J Med Sci Sports 2009 11 19 29 18282221 10.1111/j.1600-0838.2008.00774.x

[B29] Groth MV Fagt S Brondsted L Social determinants of dietary habits in Denmark Eur J Clin Nutr 2001 11 959 966 10.1038/sj.ejcn.1601251 11641744

[B30] Møller A Saxholt E Levnedsmiddeltabeller, 4. udgave (Danish food composition tables, The composition of foods - 4th edition) 1996 Ministry of Health, National Food Agency, Søborg

[B31] Gibson RS Measurement errors in dietary assessment Principles of Nutritional Assessment 1990 New York: Oxford University Press 85 96

[B32] Brug J Tak NI te Velde SJ Bere E de B I Taste preferences, liking and other factors related to fruit and vegetable intakes among schoolchildren: results from observational studies Br J Nutr 2008 11 Suppl 1 S7 S14 18257952 10.1017/S0007114508892458

[B33] Cooke LJ Wardle J Age and gender differences in children's food preferences Br J Nutr 2005 11 741 746 10.1079/BJN20051389 15975175

[B34] Bere E Brug J Klepp KI Why do boys eat less fruit and vegetables than girls? Public Health Nutr 2007 11 321 325 10.1017/S1368980007000729 17666125

[B35] Caine-Bish NL Scheule B Gender Differences in Food Preferences of School-Aged Children and Adolescents J Sch Health 2009 11 532 540 10.1111/j.1746-1561.2009.00445.x 19840230

[B36] Fisher JO Birch LL Eating in the absence of hunger and overweight in girls from 5 to 7 y of age Am J Clin Nutr 2002 11 226 231 12081839 10.1093/ajcn/76.1.226 PMC2604807

[B37] Wardle J Carnell S Cooke L Parental control over feeding and children's fruit and vegetable intake: how are they related? J Am Diet Assoc 2005 11 227 232 10.1016/j.jada.2004.11.006 15668680

[B38] Liem DG Mars M de Graaf C Sweet preferences and sugar consumption of 4- and 5-year-old children: role of parents Appetite 2004 11 235 245 10.1016/j.appet.2004.05.005 15527925

[B39] Jansen E Mulkens S Jansen A Do not eat the red food!: prohibition of snacks leads to their relatively higher consumption in children Appetite 2007 11 572 577 10.1016/j.appet.2007.03.229 17490786

[B40] Lewis MK Hill AJ Food advertising on British children's television: a content analysis and experimental study with nine-year olds Int J Obes Relat Metab Disord 1998 11 206 214 10.1038/sj.ijo.0800568 9539187

[B41] Chapman K Nicholas P Supramaniam R How much food advertising is there on Australian television? Health Promot Int 2006 11 172 180 10.1093/heapro/dal021 16835276

[B42] Harrison K Marske AL Nutritional Content of Foods Advertised During the Television Programs Children Watch Most Am J Public Health 2005 11 1568 1574 10.2105/AJPH.2004.048058 16118368 PMC1449399

[B43] Neville L Thomas M Bauman A Food advertising on Australian television: the extent of children's exposure Health Promot Int 2005 11 105 112 10.1093/heapro/dah601 15722367

[B44] Kelly B Smith B King L Flood V Bauman A Television food advertising to children: the extent and nature of exposure Public Health Nutr 2007 11 1234 1240 17381920 10.1017/S1368980007687126

